# Hybrid Target Selections by ”Hand Gestures + Facial Expression” for a Rehabilitation Robot

**DOI:** 10.3390/s23010237

**Published:** 2022-12-26

**Authors:** Yi Han, Xiangliang Zhang, Ning Zhang, Shuguang Meng, Tao Liu, Shuoyu Wang, Min Pan, Xiufeng Zhang, Jingang Yi

**Affiliations:** 1 The State Key Laboratory of Fluid Power and Mechatronic Systems, School of Mechanical Engineering, Zhejiang University, Hangzhou 310027, China; 2 Department of Intelligent Mechanical Systems Engineering, Kochi University of Technology, 185 Miyanokuchi, Tosayamada-Cho, Kochi 782-8502, Japan; 3 Key Laboratory of Rehabilitation Technical Aids Technology and System of the Ministry of Civil Affairs, National Research Center for Rehabilitation Technical Aids, Beijing 100176, China; 4 Faculty of Information Engineering and Automation, Kunming University of Science and Technology, Kunming 650000, China; 5 Department of Mechanical Engineering, University of Bath, Bath BA2 7AY, UK; 6 Department of Mechanical and Aerospace Engineering, Rutgers University, Piscataway, NJ 08854, USA

**Keywords:** facial expression, hybrid control gestures, interactive tasks, rehabilitation robot, target selection

## Abstract

In this study we propose a “hand gesture + face expression” human machine interaction technique, and apply this technique to bedridden rehabilitation robot. “Hand gesture + Facial expression” interactive technology combines the input mode of gesture and facial expression perception. It involves seven basic facial expressions that can be used to determine a target selecting task, while hand gestures are used to control a cursor’s location. A controlled experiment was designed and conducted to evaluate the effectiveness of the proposed hybrid technology. A series of target selecting tasks with different target widths and layouts were designed to examine the recognition accuracy of hybrid control gestures. An interactive experiment applied to a rehabilitation robot is designed to verify the feasibility of this interactive technology applied to rehabilitation robots. The experimental results show that the “hand + facial expression” interactive gesture has strong robustness, which can provide a novel guideline for designing applications in VR interfaces, and it can be applied to the rehabilitation robots.

## 1. Introduction

Gestures and facial expressions have been the focus of a lot of recent research. They offers a compelling platform for touchless interactions. The advantages and applications of face detection are well-explored in the literature [1,2,3,4,5,6,7,8]. The advantages of facial expressions including the Face Switch have been well documented and investigated in research [9]. It’s a device that can interact with a computer through a combination of facial expressions and gestures.

**Figure 1:**
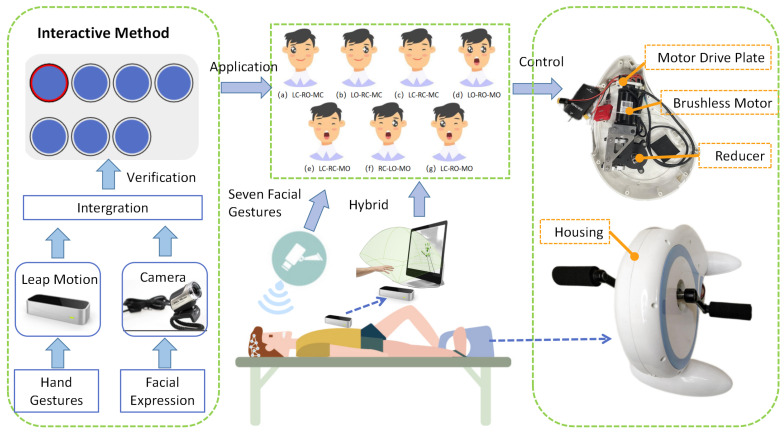
Hybrid Target Selections by “Hand Gestures + Facial Expression” for a Rehabilitation Robot.

In this paper, a new computer interactive hybrid gesture control technique is proposed. User facial expression data and gesture data are collected based on the camera and Leap Motion [10,11,12], providing a new input dimension for interaction. This design realizes the combination of facial expressions and gestures, the selection of targets and the control of rehabilitation robots and other operations (Figure 1).

The development of face detection technology based on artificial intelligence provides a technical basis for the exploitation of facial expressions. Pilarczyk et al. [3] proposed a computer algorithm based on a CNN/MMD face detector that recognizes human emotions through network cameras. Chu and Peng [7] explored the user identification problems associated with facial recognition technology to unlock mobile devices. Kim and Cha [13] proposed a hands-free natural user interface, which is a facial expression study based on a head-mounted display (HMD) sensor, and users need to wear an HMD device. By contrast, our research does not require users to wear any equipment, thus increasing user comfort versatility. David et al. [9] proposed a FaceSwitch accessibility software system designed to provide facilitated computer interaction for users with compromised upper limb mobility. This software uses a deformable face tracker to track the landmark features of the user’s face. This system enables the user to map a specific facial expression to custom computer control commands that interact with the computer based on a camera and an eye tracker.

Florian et al. [14] proposed a way to trigger interaction with the contours of a medium object using a gaze gesture. Zhang, Harish, and Kulkarni [15] proposed a gaze peak application that could explain eye gestures in real-time. These gestures were decoded into predictive movements and provided the users with a non-denying interface to facilitate user communication. Sun et al. [16] proposed a spatiotemporal fusion model and multi-mode hierarchical fusion strategy. Li et al. [17] proposed an HRI model of a robot arm based on gesture and body motion recognition algorithms. Al-Hammadi et al. [18] proposed an effective deep convolutional neural network method for gesture recognition. Anja and Gebhard [19] conducted a usability study employing head movements and head poses to aid disabled people who cannot use their upper limbs. Garcia et al. [20] used body gestures to navigate interactive maps.

Many researchers have applied gesture to rehabilitation robots [21,22,23,24]. Segal et al. [22] presented the design and testing of a gesture-controlled rehabilitation robot (GC-Rebot) to estimate its potential for monitoring user performance and providing entertainment while conducting physical therapy. Michael et al. [25] presented a new gesture-based human interface for natural robot control. RongWen et al. [26] proposed a cooperative surgical robot system, guided by hand gestures and supported by an augmented reality (AR)-based surgical field, for robot-assisted percutaneous treatment. Geng et al. [27] proposed a novel gesture recognition system for intelligent interaction with a nursing-care assistant robot. Marek et al. [28] applied body posture tracking devic to rehabilitation robot. Jessica et al. [29] presented the development of an interactive virtual reality (VR)-based framework that allows one to simulate the execution of rehabilitation tasks and robotic assistance through a robotic standing wheelchair. Fusco et al. [30] study indicates how the combination of robotic treatment with VR is effective in enhancing the recovery of cognitive function in patients with ABI, also improving disability and muscular function. Feng et al. [31] presented a dual-modal hybrid self-switching control strategy automatically determine the exercise mode of patients, i.e., passive and assistive exercise mode. Dong et al. [32] developed three different rehabilitation training methods adapt to the patients at different stages of rehabilitation, namely, passive exercise, active exercise and resistance exercise, respectively. Prieto et al. [33] designed a wearable immersive virtual reality device for promoting physical activity in parkinson’s disease patients. Sun et al. [34] proposed a facial emotion recognition system for exoskeleton rehabilitation robot. Bien et al. [35] developed a rehabilitation robotic system with various human-robot interaction interfaces for the disabled.

There are already a number of rehabilitation robots for lower limbs on the market, which can be divided into standing, multi-posture and sitting and horizontal according to different training posture. The standing lower limb rehabilitation robot is mainly divided into two categories, one is the hanging rehabilitation robot represented by Lokomat [36] developed by Hocoma company in Switzerland, the other is the wearing lower limb rehabilitation robot represented by Elegs [37] developed by Berkeley Company in the United States. The multi-type lower limb rehabilitation robot mainly adopts the rehabilitation strategy combining the standing lower limb rehabilitation robot and standing bed. In the aspect of sitting-horizontal rehabilitation robot, the Motion Maker lower limb rehabilitation robot developed by SWORTEC of Switzerland has been put into clinical application [38].

In summary, these technologies have enriched the pool of multi-channel human-computer interactions. The interactive technology in virtual space (meta universe) needs to be explored. However, few study has yet combined facial expressions and hand gestures; thus, inspiring our foray into hybrid gestures technology. The hybrid control gestures technology presented in this study developed a new type of interaction that combines facial expressions and hand gestures. It can receive the simultaneous interaction of hand gestures and facial expressions to perform interactive tasks. The technology provides design guidelines for VR exoskeleton-based interactions.

The accessibility of the hybrid control gestures technique with the following features is explored:

- A hybrid control gestures technique with two input modalities from hand gestures and facial expression is described;
- It is a touchless interactive technique;
- It can be mapped to the interactive mode of the virtual reality environment;
- Facial expressions correspond to basic control functions;
- Hand gestures are detected by Leap Motion, providing a virtual hand technique for cursor movement control.

This paper is organized as follows. Section 2 describes the optimization method, software platform, and hardware equipment of hybrid control gesture technology. In Section 3, the empirical analysis of hybrid control surgery technology is carried out and the application of interactive technology in upper limb rehabilitation robots. In Section 4, the experimental results and design guidelines are discussed. In Section 5, the conclusion is drawn.

## 2. Materials and Methods

### 2.1. Interactive Method

#### 2.1.1. Interactive Hardware

A Lenovo computer with the Windows 10 operating system was used in the experiments. A second-generation Leap Motion sensor was used in the experiments to detect hand movements. The Leap Motion sensor was placed 15 cm from the center edge of the monitor base. The external camera, an ANC Core HD 1080p-Y3 webcam, was used to provide a continuous image stream of the subject’s face to a face tracking application described later. It was located at the top of the monitor, 8 cm from the left edge of the display. This distance was only set for consistency of the experimental conditions to reduce deviation resulting from different distances.

#### 2.1.2. Seven Facial Gestures

**Figure 2:**
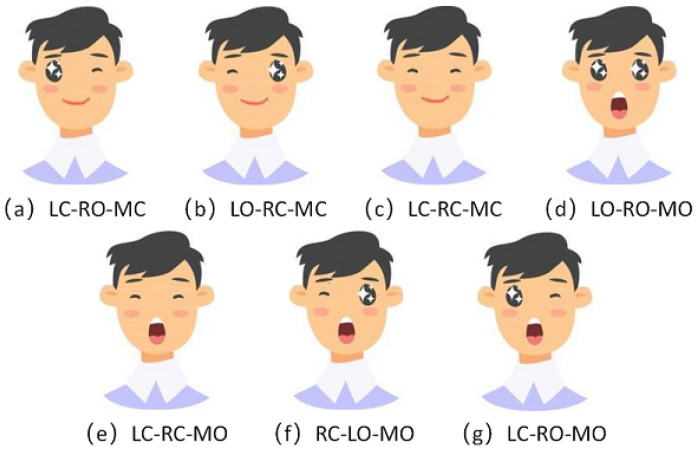
Facial gestures and commands: (**a**) Closing of the left eye; Opening of the right eye; Closing of the mouth. (**b**) Opening of the left eye; Closing of the right eye; Closing of the mouth. (**c**) Closing of the left eye; Closing of the right eye; Closing of the mouth. (**d**) Opening of the left eye; Opening of the right eye; Opening of the mouth. (**e**) Closing of the left eye; Closing of the right eye; Opening of the mouth. (**f**) Closing of the right eye; Opening the left eye; Opening of the mouth. (**g**) Closing of the left eye; Opening of the right eye; Opening of the mouth.

Seven facial expressions were defined according to the detected facial features, as shown in Figure 2.

#### 2.1.3. Software and Method

Hand capture software was used for hand interaction purposes. This software provided gestural information to track the user’s hand movements while interacting with the computer. The user activated a task by continuously moving his/her hands. Subsequently, the user could move a cursor using his/her hands at any location on the screen.

A camera was used to track the user’s face while interacting with the computer. The camera tracked several specific facial features, such as the opening and closing of the mouth and the opening and closing of the eyes. The facial expression recognition system was used to monitor facial expressions in real-time and to correspond them to specific control commands. Our software provided a friendly and natural way to customize and chart specific gestures to different mouse events.

The interactive zone of Leap Motion was a box. Provided the user’s hand or finger was within this box, it could be tracked by Leap Motion. Because there is a certain amount of jitter in the direct control of the mouse movement through Leap Motion, the Kalman filter [39] method is used to control the jitter, and the data collected by Leap Motion and the cursor state data at the previous moment are used to generate the current cursor state through the Kalman filter method [36]. To avoid the influence of noise caused by Leap Motion’s sensor noise and hand jitter.

The Kalman filter uses the cursor position state at the previous moment and the instantaneous velocity at the current moment to predict the a priori estimate of the cursor state at the current moment X${}_{t}$’:

(1) $$X_{t}{}^{\prime }=X_{t-1}+{aU}_{t}+W_{t}$$

X${}_{t-1}$ is the cursor position state at the previous moment; U${}_{t}$ is the instantaneous speed of the system; Wt is hand jitter noise; a is the time interval Delta t.

And the process error covariance Pt’ formula of the synchronization update is:

(2) $$P_{t}^{\prime }=bP_{t-1}+Q_{\prime }$$

Q is hand jitter noise variance. Then calculate the Kalman gain K${}_{t}$:

(3) $$K_{t}=P_{t}^{\prime }/P_{t}^{\prime }+R$$

R is the sensor noise variance.

At the same time, the data obtained by Leap Motion is used to modify the value of the prior estimation to achieve the optimal estimation of the cursor state at the current moment X${}_{t}$:

(4) $$X_{t}=X_{t_{m}^{\prime }}+K_{t}\left(Z_{t}-X_{t^{\prime }}{}^{\prime }\right)$$

Z${}_{t}$ is the status data collected by Leap Motion.

The updated observation noise covariance Pt formula is:

(5) $$P_{t}=\left(1-K_{t}^{\prime }\right)P_{t}^{\prime }$$

**Figure 3:**
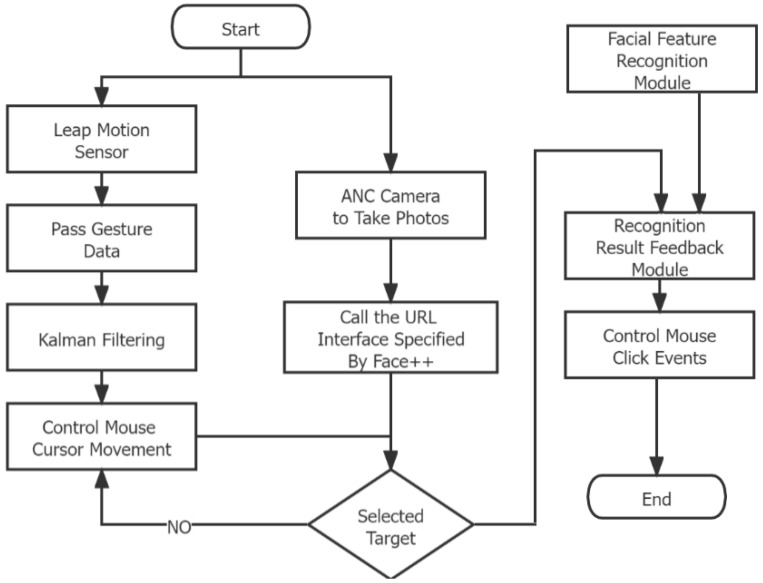
Flow chart of hybrid gesture control.

Figure 3 describes the operation of the program. The system began to divide into two bifurcations, the hand and the face. In the hand system, first, the Leap Motion sensor is activated by the hand. Obtain hand information, and through Kalman filtering, the mouse cursor can be stably controlled by the movement of the hand. In the face system, the ANC camera obtains facial expressions and performs facial expression recognition through Face++. Participants move the mouse cursor with their hands to select the experimental target. After the target is selected, the system analyzes the facial expression data recognized by Face++, and click the event to complete.

### 2.2. Rehabilitation Robot

Using the interaction method proposed above, the present work designed and developed a lower limb rehabilitation robot based on Hand Gestures + Facial Expression, which includes two training modes: active training mode and passive training mode. It is mainly used for bed rehabilitation training of patients with insufficient muscle strength in the early stage of rehabilitation. The present work switched between training modes based on gestures. In the active training mode, the resistance was adjusted according to the facial expression. In passive training mode, adjust the speed based on facial expressions.

#### 2.2.1. Rehabilitation Robot Hardware

**Figure 4:**
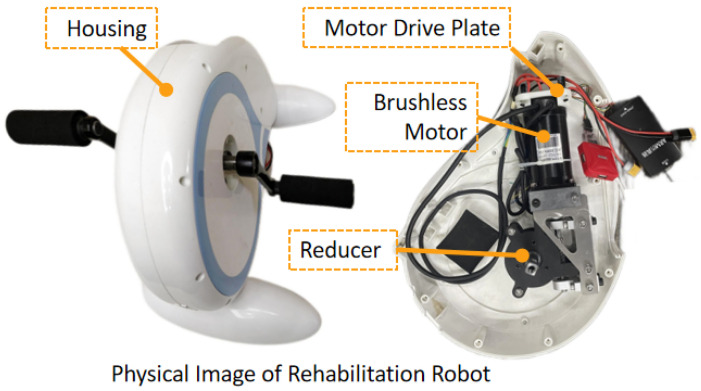
The prototype’s image of the proposed rehabilitation robot.

The lower limb rehabilitation robot developed at present uses the sensor FOC algorithm to drive the permanent magnet synchronous motor. The main control MCU selects STM32G431RBT6 chip from ST company, and the motor driver selects DRV8323RS three-phase grid driver chip from TI company. The motor Angle is obtained by TLE5012BE1000 magnetic encoder. In addition, it also includes a dissipation circuit module, 24 V power supply module, ADC sampling circuit module, and interface module. After the motor drive plate and worm gear reducer was connected, place them in the existing housing of rehabilitation devices, and install the handle and baseas shown in Figure 4.

#### 2.2.2. Rehabilitation Robot Software

The software of the lower limb rehabilitation robot is divided into a lower computer driver and upper computer control program. The lower computer driver mainly drives the permanent magnet synchronous motor. The development language is C language. The upper computer control program mainly includes the functions of switching training mode, visualizing the training state, training data, etc. The development language is Python, and the third-party libraries used are pyQt5 and pygame. The upper computer control program switches between different training modes and training stops based on gesture, adjusts the resistance based on facial expression in active training mode, and adjusts the rotation speed based on facial expression in passive training mode. There are three gears in the active training mode. The torque of gear A is 15 N and activated by LO-RO-MO expression; the torque of gear B is 30 N and activated by LC-RC-MO expression; and the torque of gear C is 45 N and activated by LC-RC-MC expression. The passive training mode is also divided into three gears. Gear A is activated by LO-RO-MO expression when the speed is 15 rpm, gear B is activated by LC-RC-MO expression when the speed is 30 rpm, and gear C is activated by LC-RC-MC expression when the speed is 50 rpm.

## 3. Experiments and Results

### 3.1. Participants

The present work designed and conducted two experiments. Experiment 1 verifies the interaction method proposed above, and experiment 2 verifies the feasibility of applying the proposed new interaction method to the lower limb rehabilitation robot. Ten participants (6 males and 4 females) were recruited from our laboratory for two experimental tests. All participants were in good health and had experience interacting with facial gesture software.

All subjects gave their informed consent for inclusion before they participated in the study. The study was conducted in accordance with the Declaration of Helsinki, and the protocol was approved by the Ethics Committee of Zhejiang University (2021-39).

### 3.2. Task and Procedure

#### 3.2.1. Experiment 1

In this experiment, six sets of circular buttons with differently-sized radii were set. These radii were 10 pixels, 15 pixels, 20 pixels, 25 pixels, 30 pixels, and 35 pixels in size. Each set of target buttons was then placed at three different heights, 6.5 cm, 16 cm, and 26.5 cm, above the target button interface. Height refers to the distance from the top of the target interface to the bottom of the monitor screen. Eighteen sets of tests were completed by placing buttons with different radii at three different heights (i.e., three heights with six radii).

Each participant performed 18 different experiments. In the experiments, target selection and facial recognition were performed according to the order of the buttons from left to right in a sequence. The desired target required a land-on approach, which meant selecting a target directly below the cursor.

The sequence of the facial expressions corresponding to the seven buttons was as follows: LO-RC-MC, LC-RO-MC, RC-LO-MO, LO-RO-MO, LC-RC-MC, LC-RC-MO, LC-RO-MO. (The sequences of the buttons were: from left to right, from top to bottom.)

Before the experiments are performed, the flow of the experiment was explained in detail to the participants. The participants were allowed 10 practice trials.

**Figure 5:**
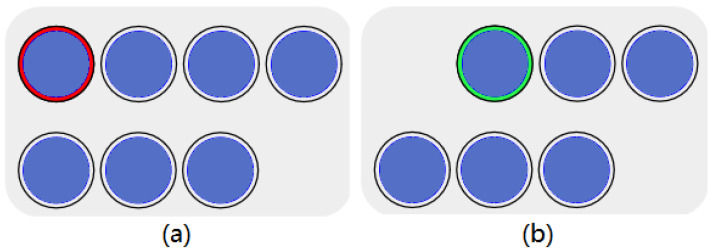
Experimental interface: (**a**) When the wrong target button is selected, the outer circle of the button is red; (**b**) The outer circle of the next target button to be recognized is green.

As shown in Figure 5, when participants moved the cursor by their hand gestures to select the target, a red circle around the target appeared. A peripheral red circle defined a wrong selection. The blue circle itself defined the correct selection area. During the experiment, participants attempted to place the cursor in the blue area of the target button.

The corresponding facial expression would be recognized only after the correct button was identified by the cursor. After successful recognition, if the cursor selected the red peripheral circle, the backstage data recorded an error in the selection information. When the cursor selected the blue area, the backstage data recorded the correct selection information. The target button disappeared after the cursor selection was completed and the facial recognition was successful. A green peripheral circle would then surround the subsequent target. This green circle directed the participants to the next target button.

Each participant needed to conduct 18 sets of experiments in the “hands + facial expressions” interactive mode. There were seven target buttons in each set of experiments and each target button corresponded to a facial expression. The sequence of facial gestures corresponding to each target button in each group of experiments was the same.

The overall design of the experiment included the following:

- Ten participants;
- Six target radii;
- Three target heights;
- Seven target buttons (In each group);
- 1260 overall target selections.

Every new interactive technology needs to be measured by human-computer interaction. The proposed work detected the accuracy of target selection through 3 s time pressure (Restrict the target selection task to be completed within 3 s, uncompleted will be regarded as a failure).

#### 3.2.2. Experiment 2

In this experiment, gestures were used to set the switch of two training modes and the emergency stop switch. The left side is the active training mode, and the right side is the passive training mode. As long as gestures are recognized during the training process, emergency stop is indicated. There are three gears in the active training mode. This experiment used three facial expressions to switch gears. The torque of gear A is 15 N, which is activated by LO-RO-MO expression, the torque of gear B is 30 N, which is activated by LC-RC-MO expression, and the torque of gear C is 45 N, which is activated by LC-RC-MC expression. The passive training mode is also divided into three gears. Similarly, used three facial expressions to switch gears. The speed of gear A is 15 rpm, which is activated by LO-RO-MO expression, the speed of gear B is 30 rpm, which is activated by LC-RC-MO expression, and the speed of gear C is 50 rpm, which is activated by LC-RC-MC expression.

**Figure 6:**
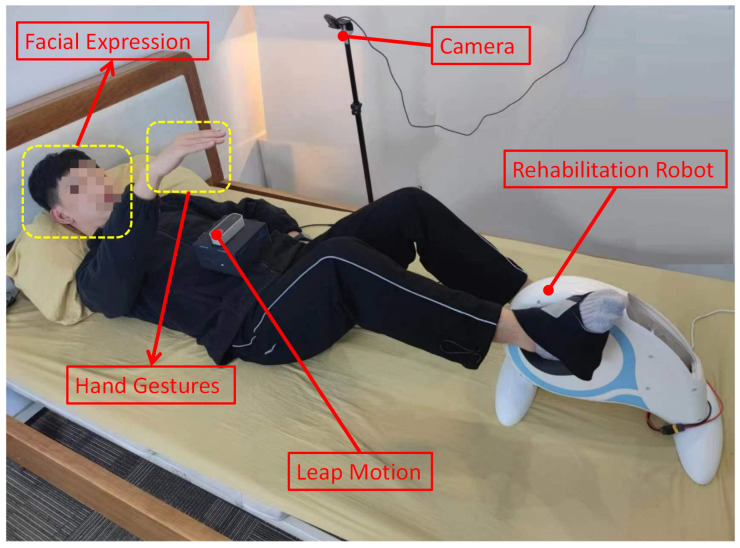
The experimental scene.

The experimental process was divided into two steps. The first step was to use the gesture selection training mode, and the experimenter needed to complete the gesture selection within 3 s. The second step is to use facial expressions to switch training gears or use gestures to stop in an emergency. The specific task of switching gears is shown in Table 1. The proposed work have preset the training mode and gear switching process in advance, and recorded whether the experimenter made the correct choice in real time during the experiment. Experimenter and experiment 1 are the same, each experimenter needs to carry out 10 sets of experiments, each set of experiments includes two mode selection, 12 gear changes in active mode and 12 gear changes in passive mode. Before the experiment began, the participants were given a detailed explanation of the experiment process and were allowed to conduct three practical experiments. The experimental scene is shown in Figure 6.

### 3.3. Results

#### 3.3.1. Experiment 1

To analyze the experimental results, target radius (10 pixels, 15 pixels, 20 pixels, 25 pixels, 30 pixels, 35 pixels) and target height (low: 6.5 cm, between 16 cm, high: 26.5 cm) were used as independent variables. A two-way repeated measures ANOVA (Analysis of Variance) was applied to determine the effect of target button radius and target height variation on the accuracy of the participant to perform the hybrid control gesture recognition. Through studentized residuals and a Shapiro-Wilk test, five groups’ data were determined not to be normally distributed; the remaining 13 groups’ data were normally distributed (*p* > 0.05).

A one-way repeated measures ANOVA was also applied to analyze whether the data were normally distributed, which proved a more robust indicator of deviation from the normal distribution, especially if the sample size of each group was equal or almost equal. Moreover, a non-normal distribution did not substantially impact the probability of making a type I error; therefore, the data could still be tested directly. Determined by studentized residuals and a standard deviation of 3, there were no outliers in any data set. The interaction term height radius (X2 = 62.262, *p* = 0.208) was obtained from the result of Mauchly’s sphericity test when it met Mauchly’s spherical assumptions; therefore, the variance-covariance matrix of the dependent variable was equal (*p* = 0.208).

The results were represented as the average standard deviation. After analysis, the interaction between height and radius and the difference in the accuracy of hybrid control gesture recognition was not statistically significant (F5.058, 349.003 = 0.988, *p* = 0.426). Therefore, the influence of the two subjects’ internal factors (height and radius) was validated.

The main effect of target height on hybrid control gestures recognition error rate was not statistically significant (F2,138 = 0.042, *p* = 0.959).

**Figure 7:**
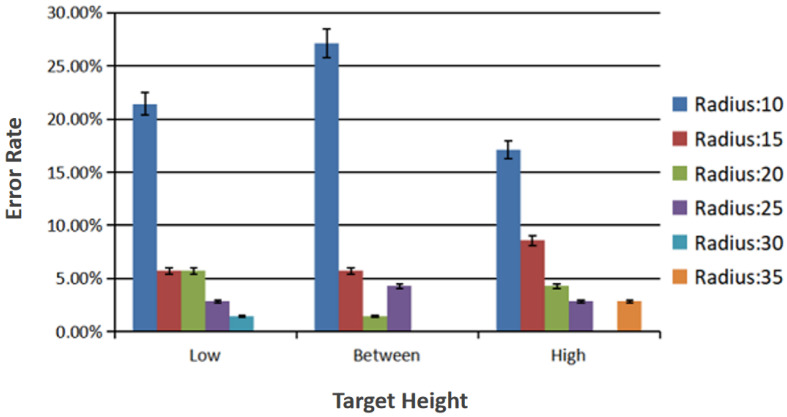
Recognition error rates for different target radii at different target heights.

The effect of the target radius on the accuracy of recognition of the hybrid control gestures was statistically significant (F5,345 = 7.841, *p* < 0.001). Since the target radius factor had six variables, pairwise comparisons were required. The difference between the hybrid control gestures recognition error rate between target radii of 10 pixels and 15 pixels was 0.152 (95% confidence interval: 0.054 0.251, *p* < 0.001), which is statistically significant. The difference between the hybrid control gestures recognition error rate of the target radii of 10 pixels and 20 pixels was 0.181 (95% confidence interval: 0.087 0.275, *p* < 0.001), which was statistically significant. The difference in the hybrid control gestures recognition error rate between the target radii of 10 pixels and 25 pixels was 0.186 (95% confidence interval: 0.094 0.277, *p* < 0.001), which was statistically significant. The difference in the hybrid control gestures recognition error rate of target radii of 10 pixels and 30 pixels was 0.214 (95% confidence interval: 0.122 0.307, *p* < 0.001), which was statistically significant. The difference in the hybrid control gestures recognition error rate between target radii of 10 pixels and 35 pixels was 0.210 (95% confidence interval: 0.119 0.300, *p* < 0.001), which was statistically significant. The difference in the hybrid control gestures recognition error rate between target radii of 15 pixels and 30 pixels was 0.062 (95% confidence interval: 0.010 0.114, *p* = 0.008), which was statistically significant. The difference in the hybrid control gestures recognition error rate between target radii of 15 pixels and 35 pixels was 0.057 (95% confidence interval: 0.003 0.112, *p* = 0.032 < 0.05), which was statistically significant. The comparison result of the recognition error rate among other target radii is not statistically significant, for example, the recognition error rate between the target radius of 15 pixels and 20 pixels is not statistically significant. Figure 7 shows the recognition error rates for different target radii at different target heights.

**Figure 8:**
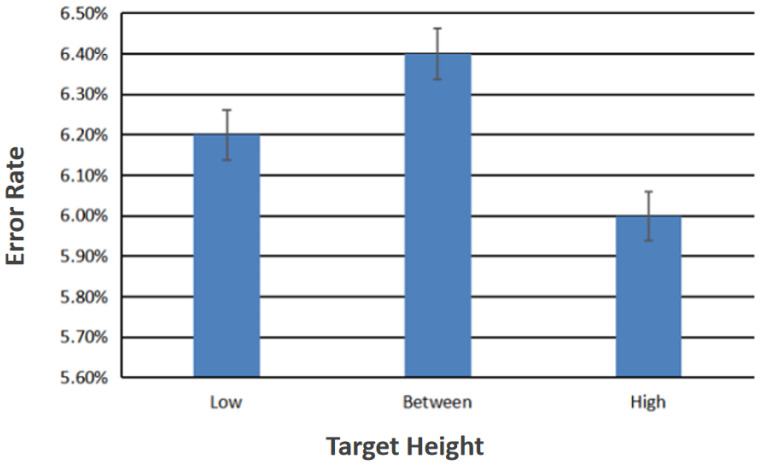
Recognition error rate different target heights.

Figure 8 shows the recognition error rate when the target radius is ignored. The ordinate represents the recognition error rate and the abscissa represents the three different target heights.

**Figure 9:**
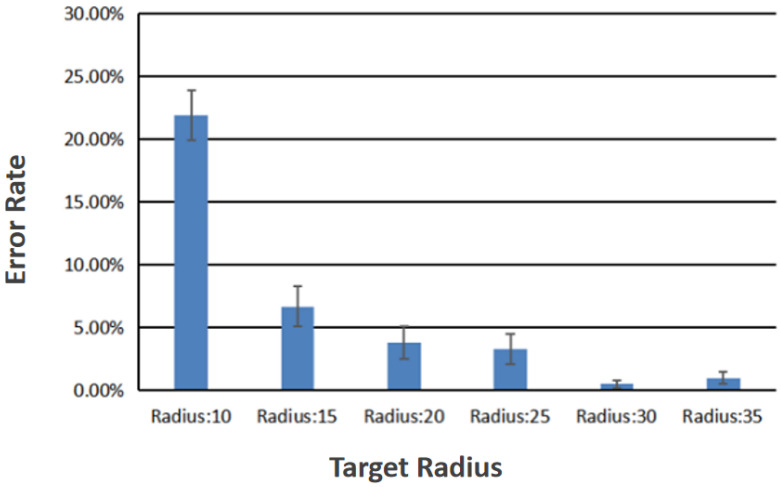
Recognition error rate different target radii.

Figure 9 shows the recognition error rate obtained when the target height is ignored. The ordinate represents the recognition error rate and the abscissa represents the six different target radii.

The data mentioned in this chapter are all accurate values obtained by the two-way repeated measure ANOVA.

The above experimental results show that “hand + facial expression” is feasible as an interaction technology. It provides data support for experiment 2 to control rehabilitation robot by gesture and facial expression.

#### 3.3.2. Experiment 2

Experiment 2 is mainly to verify the accuracy of the interactive method control of the rehabilitation robot. This experiment includes 26 subtasks in total, including 2 mode switching subtasks and 24 gear switching subtasks. Each subtask has 100 experiments in total, and each control subtask will record whether it is correctto calculate the error rate of each subtask.

**Figure 10:**
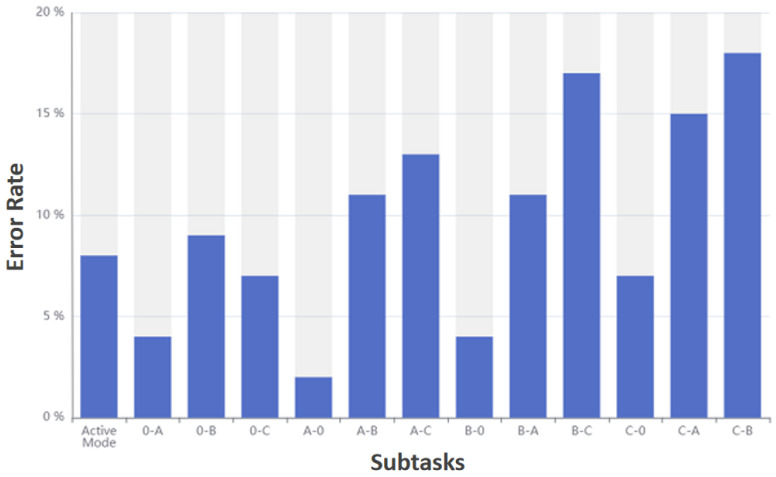
An error rate of each subtask in active mode.

Figure 10 shows the error rate of each subtask in the active mode. The ordinate identifies the error rate, and the abscissa represents different subtasks.

**Figure 11:**
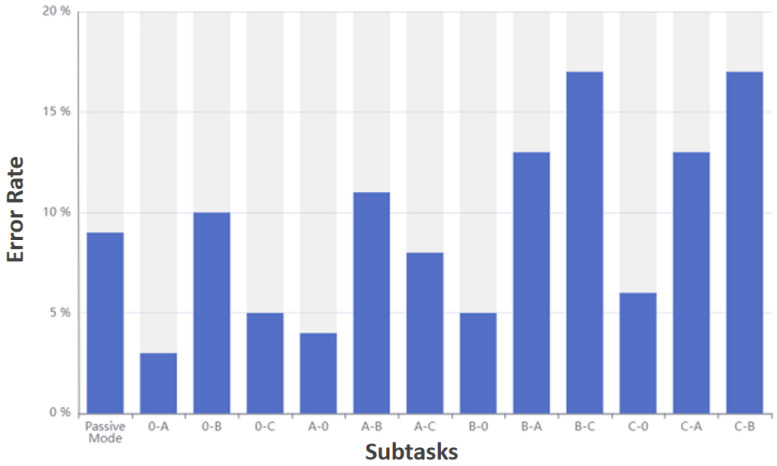
Error rate of each subtask in passive mode.

Figure 11 shows error rate of each subtask in the passive mode is displayed. The ordinate identifies the error rate, and the abscissa represents different subtasks.

Experimental results show that both modes have the highest error rate when switching between B and C. The overall error rate is lower than the selection error rate in experiment 1.

## 4. Discussion

### 4.1. Data Analysis Conclusion

In this experiment, three target heights and six target radii were tested. The differences in accuracy of the hybrid control gesture recognition under these variables were compared. From the analysis of all the data, we drew the following conclusions:

- As shown in Figure 8, if the target radius is ignored, the error rate of recognition when the target height is limited to “high” is the lowest. The error rate of recognition is the highest when the target height is limited to “between”;
- As shown in Figure 9, if the target height is ignored, the error rate of recognition when the target radius is limited to 30 pixels is the lowest. The error rate of recognition is the highest when the target radius is limited to 10 pixels.
- The interaction between height and radius and the difference in the accuracy of hybrid control gesture recognition was not statistically significant. The target height has no significant effect on the selection error rate. The size of the target radius has a significant impact on the selection error rate.

According to the above conclusions, for the target of the hybrid “hand + facial expression” gestures, the position factor of the target can be ignored, and only the target radius factor needs to be concerned. When the target radius is limited to 30 pixels and above, the interactive pattern recognition rate can reach 93.81%, which further are very robust. The present work verified the error rate of this interaction technology applied to the lower limb rehabilitation robot through experiments. The results show that this interaction technology can be applied to the rehabilitation robots, and the interaction accuracy can be improved.

### 4.2. Design Guideline

Based on the research and experience in hybrid control gestures, the interactive operation of the simulated virtual environment through the camera and Leap Motion has extremely high reference significance for the hybrid gesture control of the virtual environment. Gestures are the most intuitive body language for pointing operations, and facial expressions are used for auxiliary control. For the gesture control, the stability method of this research can be used to improve the ease of gesture control. The basic operations such as confirmation through simple and easy-to-collect facial expressions can reduce the misoperation of gestures and enhance the ease of use of gesture control. Based on the gesture control, a dimensional operation is added to achieve target selection tasks in more complex scenes.

Hybrid control gesture technology removes physical barriers, and can interact with computers in a non-contact way with the help of VR interactive exoskeletons, and even provides an alternative solution for people with disabilities to perform interactive operations. It also provides a new interactive method for solving the interactive task of target selection in the virtual space.

## 5. Conclusions

The present work design and propose a hybrid gesture target selection technology which is suitable for multiple scene interaction. The cursor movement is controlled by gestures, and facial expressions are used to confirm the operation (ie, “click”). The sensor simulates the scene to collect gesture and facial expression information and evaluates the influence of target size and target height on selection time and accuracy. The results show that the target height (position) has no significant effect on the error rate and task time, while the target radius has a significant effect on it. When the target width is greater than 30 pixels, it can reach a recognition accuracy of 93.81%, which is very robust. The results of the interaction experiments on rehabilitation robots show that the application of this interaction technology on rehabilitation robots is feasible.

## Figures and Tables

**Table 1:** Gear shift task.

Serial No	Gear Shift	Serial No	Gear Shift
1	Shift gear A to stop	7	Shift gear B to stop
2	Shift gear C to stop	8	Shift gear A to B
3	Shift gear B to A	9	Shift gear C to A
4	Shift gear A to C	10	Shift gear B to C
5	Shift gear C to B	11	Stop switching to gear A
6	Stop switching to gear B	12	Stop switching to gear C

## Data Availability

The data presented in this study are available on request from the corresponding author. The data are not publicly available due to privacy.
